# PhotoChem Reference Chemical Database for the Development of New Alternative Photosafety Test Methods

**DOI:** 10.3390/toxics13070545

**Published:** 2025-06-28

**Authors:** Ga-Young Lee, Jee-Hyun Hwang, Jeong-Hyun Hong, Seungjin Bae, Kyung-Min Lim

**Affiliations:** 1 College of Pharmacy, Graduate School of Pharmaceutical Sciences, Ewha Womans University, Seoul 03760, Republic of Korea; ryoung22@korea.kr (G.-Y.L.); cocumuk@naver.com (J.-H.H.); hongjh126@gmail.com (J.-H.H.); 2 Graduate Program in Innovative Biomaterials Convergence, Ewha Womans University, Seoul 03760, Republic of Korea

**Keywords:** phototoxic test, photosensitization test, reference chemical, alternative test method, chemical database

## Abstract

Photosafety assessments are a key requirement for the safe development of pharmaceuticals, cosmetics, and agrochemicals. Although in vitro methods are widely used for phototoxicity and photoallergy testing, their limited applicability and predictive power often necessitate supplemental in vivo studies. To address this, we developed the PhotoChem Reference Chemical Database, comprising 251 reference compounds with curated data from in vitro, in vivo, and human studies. Using this database, we evaluated the predictive capacity of three OECD in vitro test guidelines—TG 432 (3T3 NRU), TG 495 (ROS assay), and TG 498 (reconstructed human epidermis)—by comparing the results against human and animal data. Against human reference data, all three test methods showed high sensitivity (≥82.6%) and strong overall accuracy: TG 432 (accuracy: 94.2% (49/52)), TG 495 (100% (27/27)), and TG 498 (86.7% (26/30)). In comparison with animal data, sensitivity remained high for all tests (≥92.0%), while specificity varied: TG 432 (54.3% (19/35)), TG 495 (63.6% (7/11)), and TG 498 (90.5% (19/21)). TG 498 demonstrated the most balanced performance in both sensitivity and specificity across datasets. We also analyzed 106 drug approvals from major regulatory agencies to assess real-world application of photosafety testing. Since the mid-2000s, the use of in vitro phototoxicity assays has steadily increased in Korea, particularly following the 2021 revision of the MFDS regulations. Test method preferences varied by region, with 3T3 NRU and ROS assays most widely used to evaluate phototoxicity, while photo-LLNA and guinea pig tests were frequently employed for photoallergy assay. Collectively, this study provides a valuable reference for optimizing test method selection and supports the broader adoption of validated, human-relevant non-animal photosafety assessment strategies.

## 1. Introduction

Photosafety testing primarily aims to evaluate two major aspects: phototoxicity and photoallergy. In pharmaceuticals, photosafety assessment refers to an integrated process that includes photochemical characterization, nonclinical testing, and human safety information, as outlined in the ICH S10 guideline (Photosafety Evaluation of Pharmaceuticals) [1]. The objective of a photosafety evaluation is to minimize the potential risks of chemicals caused by concomitant light exposure to humans. Phototoxicity is defined as a nonimmunologic skin reaction that occurs when reactive chemicals and sunlight interact simultaneously. Such reactions occur shortly after concomitant exposure to photoreactive chemicals and light and often resemble moderate to severe sunburns. In contrast, photoallergy is an acquired immune-mediated reaction in which a photoactive substance activated by sunlight forms an allergenic hapten. This reaction is triggered by either antibody-mediated (immediate) or cell-mediated (delayed) immune responses. Unlike phototoxicity, photoallergy can occur with significantly lower energy exposure. When induced by exogenous substances, it almost exclusively manifests as delayed-type hypersensitivity (type IV). Additionally, this reaction requires an induction period before clinical symptoms appear [2]. This reaction requires a sensitization phase before clinical symptoms manifest [3]. Clinically, photoallergic reactions commonly present as erythema, pruritus, and vesicular lesions, often accompanied by skin lichenification or desquamation. These responses may resemble urticarial or lichenoid eruptions. In particular, flat-topped, keratinized lesions are a characteristic feature of such reactions.

The photoreactive potential of a chemical can be identified by evaluating its absorption in the ultraviolet (UV) spectrum. To elicit a phototoxic or photoallergic response, a chemical must exhibit sufficient light absorption (≥1000 L/mol·cm) within natural sunlight (290–700 nm) [1].

The term photosensitization is often used as a general descriptor for all light-induced tissue responses. However, to ensure clarity between phototoxicity and photoallergy, ICH S10 discourages the use of this term. Accordingly, this study will exclusively focus on the evaluation of phototoxic and photoallergic responses. When a chemical is identified as photoreactive, it is assessed using specific toxicological test methods designed to evaluate both phototoxicity and photoallergy. Key considerations for identifying a photosafety hazard include (1) light absorption within the natural sunlight spectrum (290–700 nm), (2) the generation of reactive oxygen species upon UV–visible light exposure, and (3) adequate distribution to light-exposed tissues (e.g., skin and eyes). If a compound does not meet at least one of these criteria, it is unlikely to pose a direct photosafety concern. Although indirect mechanisms may increase photosensitivity (e.g., metabolic activation), they are not currently addressed due to the lack of established test methods [3]. Currently, in vitro photosafety assessments are primarily focused on phototoxicity and ROS evaluations as specified in OECD and ICH guidelines replacing in vivo phototoxicity tests. However, photoallergy evaluation still requires in vivo experiments, as summarized in Table 1.

While in vitro photosafety tests considerably reduced the use of in vivo tests, comprehensive photosafety assessments still need in vivo tests due to the limitation of in vitro tests in the applicability domain and predictive capacity, demanding novel photosafety testing methods capable of accurately identifying chemicals that induce phototoxic or photoallergic reactions. Here, we compiled a comprehensive reference chemical database of 251 reference chemicals, including 59 phototoxic, 26 non-phototoxic, and 4 photoallergenic substances verified in humans, for the development and evaluation of alternative methods for phototoxic and photoallergy tests.

## 2. Materials and Methods

### 2.1. Construction of Phototoxicity Reference Substance Database

To support the research and validation of new phototoxicity test methods, a phototoxic reference chemical database was established. Each chemical was documented with key identifiers such as CAS numbers, physicochemical properties, and UV absorption parameters. To facilitate the development of testing methods across diverse chemical categories, information on their usage classification—pharmaceuticals, cosmetics, industrial chemicals, and agrochemicals—was also included. Both in vitro and in vivo data were collected. Importantly, test results from the OECD Test Guidelines (TGs) and human patch tests were incorporated to evaluate the predictive power and consistency between testing methodologies.

### 2.2. Phototoxicity Testing Methods Used for Pharmaceuticals

The phototoxicity testing methods used in the nonclinical stages of pharmaceutical development were investigated through searching regulatory approval documents and assessment reports from the U.S. FDA, Japan’s PMDA, the EMA, and South Korea’s MFDS.

### 2.3. Comparison of In Vivo and In Vitro Data of Phototoxicity Reference Chemicals

To evaluate the predictive performance and reliability of in vitro phototoxicity test methods, a comparative analysis was conducted using 252 reference chemicals with available in vivo or human data. The in vitro test methods were based on three internationally validated OECD guidelines (Table 2).

Only chemicals for which both in vitro and in vivo test results were available were included in the comparative analysis. Each compound was classified as either phototoxic (PT) or non-phototoxic (NPT) based on the outcomes of both test types. The sensitivity, specificity, and accuracy were calculated to assess the performance of each in vitro method.

### 2.4. Photochemical Properties

To evaluate the potential for light-induced reactivity, the primary criterion was whether the chemical absorbs light within the 290–700 nm wavelength range. A chemical was considered unlikely to cause direct phototoxicity if it exhibited a molar extinction coefficient (MEC) of less than 1000 L/mol·cm in this range. That is, if the chemical had an MEC value below 1000 L/mol·cm between 290 and 700 nm and lacked additional supportive data, it was deemed not to pose a photosafety concern in humans, according to ICH S10 guidelines [1].

## 3. Results

### 3.1. Analysis of Reference Chemicals in the PhotoChem Database

A total of 251 reference chemicals were collected and classified based on their photo-toxicity and photosensitization profiles. These compounds are widely used in various industries, including pharmaceuticals, cosmetics, and agriculture, where photoreactivity under ultraviolet (UV) exposure plays a significant role in safety evaluation.

For each chemical, various physical properties were recorded, including physical state; molar absorbance (ε); maximum UV absorption wavelength (λ_max_); and test results across human, animal, and in vitro models.

To better understand the material characteristics, the physical states of all 251 chemicals were analyzed. As shown in Table 3, the majority of the chemicals were in solid form (*n* = 218, 86.9%), followed by liquids (*n* = 26, 10.4%). Gels accounted for two chemicals, and viscous or oil-based chemicals, categorized as “Other,” also accounted for two chemicals (0.8%), while three chemicals were labeled as “Unknown” due to the absence of a definitive classification (1.2%).

### 3.2. Human vs. Animal Test Results for Reference Test Chemicals

**Figure 1:**
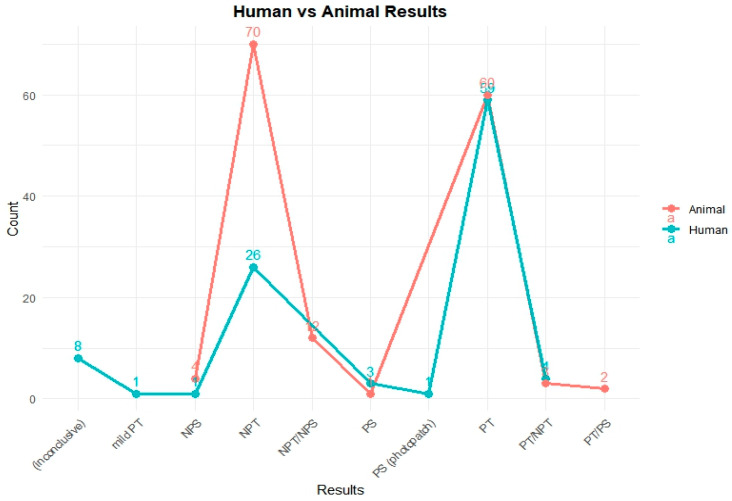
Comparison of photosafety results from human and animal tests in the PhotoChem DB. This figure shows the photosafety results for reference chemicals based on human and animal test data. In animal studies, most compounds were clearly categorized as either non-phototoxic (NPT) or phototoxic (PT). In contrast, human data included a broader range of outcomes such as inconclusive results, mild reactions, or photosensitization (photopatch), reflecting the complexity of clinical assessments.

To assess the phototoxic potential of the reference chemicals in human models, available in vivo test data were categorized by their respective outcomes. Figure 1 illustrates the comparative distribution of test results from human and animal studies for 251 chemicals.

A total of 103 chemicals out of the PhotoChem DB have human test results: 59 chemicals were identified as phototoxic (PT); 26 chemicals were categorized as non-phototoxic (NPT); 8 chemicals were reported as inconclusive; 4 chemicals were given a PT/NPT; 3 chemicals were labeled as photosensitive (PS); and a single case each was observed for mild phototoxicity (mild PT), non-photosensitization (NPS), and PS (photo patch) testing, with each indication a specialized or rare response type.

Animal model studies were also reviewed to evaluate the phototoxicity potential of the 251 reference chemicals. These models provide complementary data for human studies and are particularly important when human testing is ethically or practically limited.

As depicted in Figure 1, the animal testing results reveal the following distribution: 70 chemicals were classified as non-phototoxic (NPT), 60 chemicals showed a positive phototoxicity (PT), 12 chemicals received mixed classifications (NPT/NPS), 4 chemicals were marked as non-photosensitive (NPS), 3 chemicals were classified as PT/NPT, 2 chemicals were classified as PT/PS, and 1 chemical was confirmed as photosensitive (PS).

### 3.3. Correlation of Human Test Data with In Vivo or In Vitro Tests

The classification results for phototoxicity and photosensitization from human and animal studies showed substantial agreement, although some variation in sensitivity was observed. Out of 251 total chemicals, those lacking paired results across test types were excluded from the analysis. Sensitivity, specificity, precision, and accuracy were evaluated accordingly (Table 4 and Table 5).

Table 5 presents the predictive performance of three OECD test methods (TG 432 (3T3 Neutral Red Uptake phototoxicity test), TG 495 (Reactive Oxygen Species assay), and TG 498 (Reconstructed Human Epidermis model)) against human or animal tests. These results indicate that while the classification of non-phototoxic chemicals aligns with some in vivo data, inconsistencies across untested or conflicting chemicals (e.g., PT and PT/NPT) highlight the need for comprehensive and standardized in vivo testing to improve the reliability of non-phototoxic classifications.

Overall, the findings emphasize the importance of integrating both human and animal data to achieve more accurate and reliable assessments of phototoxicity and photosensitization. Leveraging the strengths of each model can enhance predictive performance and improve overall safety evaluation strategies.

### 3.4. Analysis of Photosafety Testing Used in Drug Approvals

In recent decades, phototoxicity and photosensitization testing have gained increasing importance in pharmaceutical safety assessments. To examine how these tests are implemented in regulatory practice, we analyzed drug approvals from major regulatory agencies including the United States Food and Drug Administration (U.S. FDA), Japan’s Pharmaceuticals and Medical Devices Agency (PMDA), the European Medicines Agency (EMA), and South Korea’s Ministry of Food and Drug Safety (MFDS). Based on compiled data from approved drug dossiers, we assessed the frequency, testing strategies, and adoption trends of photosafety evaluations.

The analysis revealed a growing adoption of photosafety testing across all agencies. In particular, data from Korea’s MFDS showed that photosafety test results were not consistently included in approval documents prior to the revision of its regulatory guidelines on 11 November 2021. At that time, certain nonclinical safety assessments, including photosafety tests, were exempted for specific categories of drugs. However, following the regulatory revision, phototoxicity and photosensitization testing became mandatory components of nonclinical safety evaluations. This regulatory shift highlights the increasing importance placed on photosafety assessments in Korea and aligns with the global trend toward comprehensive photoreactivity risk management.

**Figure 2:**
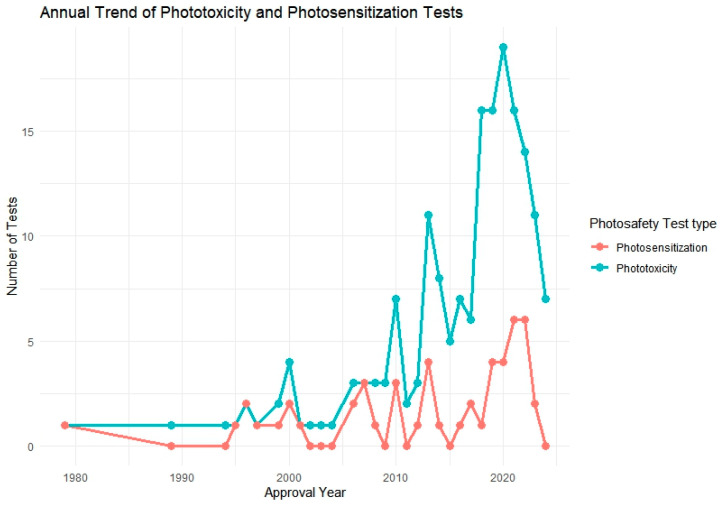
Annual trend of phototoxicity and photosensitization testing among approved pharmaceutical products (n = 106). The graph illustrates the increasing frequency of photosafety testing over the years, particularly for phototoxicity since the mid-2000s.

**Figure 3:**
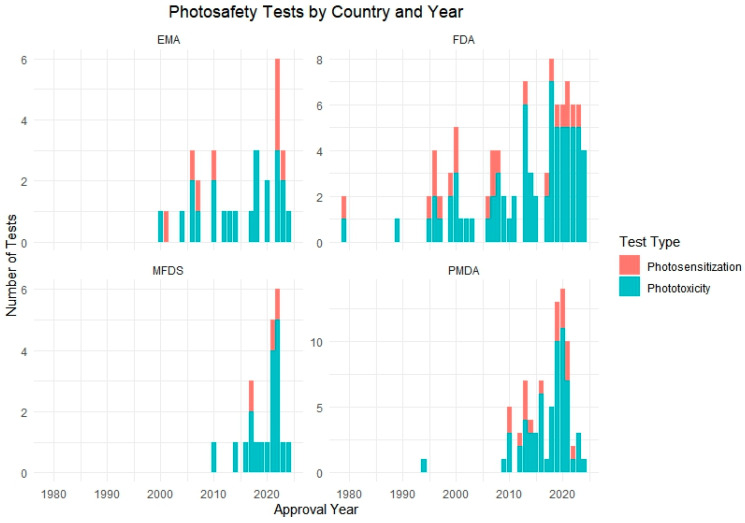
Number of phototoxicity and photosensitization tests conducted by country and year. The chart displays the test method distribution across four regulatory authorities: the FDA, PMDA, EMA, and MFDS.

Following this, we further analyzed a total of 106 approved pharmaceutical products that included photosafety assessments, focusing on their annual adoption patterns and the specific test methods used. To visualize this trend, we generated a yearly distribution graph showing the implementation of phototoxicity and photosensitization testing over time (Figure 2). In addition, test method utilization was analyzed by regulatory authority, including the FDA, PMDA, EMA, and MFDS, to assess differences in test preferences among countries (Figure 3).

The analysis revealed a notable increase in the application of phototoxicity tests beginning in the mid-2000s, followed by a gradual rise in photosensitization testing in more recent years. Regionally, the PMDA and FDA showed the highest number of total test submissions, while MFDS data showed a steep increase post-2021. Across agencies, 3T3 NRU, ROS assays, and RHE-based models were most commonly used for phototoxicity, whereas LLNA and guinea pig-based protocols were predominantly used for photosensitization.

Figure 2 illustrates the annual trend in phototoxicity and photosensitization testing from 1979 to 2024, based on data extracted from 106 approved pharmaceutical products that included photosafety evaluations. During the early years of the dataset, reports of both phototoxicity and photosensitization testing were infrequent or absent, likely due to the absence of standardized regulatory requirements or test guidelines during that time.

A notable shift occurred beginning in the mid-2000s, with a steady increase in phototoxicity test implementation. This upward trend culminated between 2018 and 2021, during which over 18 phototoxicity assessments were recorded in a single year. This surge may be attributed to the widespread regulatory adoption of OECD TG 432 and increased emphasis on nonclinical photosafety data in global drug development and review processes.

In contrast, photosensitization testing exhibited a more gradual increase, with a relatively small number of cases—typically fewer than five per year—reported since 2010. This modest rise suggests that photosensitization assessments are applied more selectively, potentially limited to compounds with specific structural alerts, photoreactive moieties, or UV absorption profiles. The overall pattern reflects a growing awareness of photosafety considerations and regulatory alignment over time, particularly for phototoxicity.

Figure 3 presents a cumulative analysis of photosafety testing conducted by regulatory authority across four countries: the United States (the FDA), Japan (the PMDA), the European Union (the EMA), and South Korea (the MFDS). The data shows a distinct pattern in how each country has adopted and implemented phototoxicity and photosensitization testing in their regulatory processes over time.

The PMDA and FDA demonstrated relatively early adoption, with consistent test submissions reported since the mid-2000s. In contrast, data from the EMA showed a gradual increase, reflecting more selective application based on compound characteristics or risk assessment strategy. The most prominent change was observed in Korea’s MFDS, where a sharp rise in test submissions occurred after 2021, coinciding with the regulatory amendment that made photosafety testing mandatory for new drug applications.

This country-specific breakdown illustrates differing timelines and regulatory emphases in adopting photosafety tests, highlighting the importance of harmonized global guidelines and the evolving role of photoreactivity evaluation in drug approval pathways.

**Figure 4:**
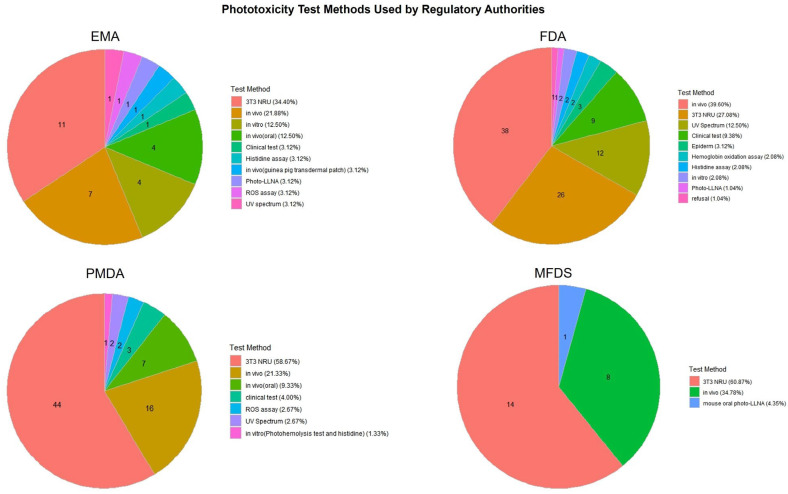
Distribution of phototoxicity test methods used in pharmaceutical approvals by country. The chart compares the types of phototoxicity test methods adopted by four major regulatory authorities (the EMA, FDA, PMDA, MFDS), illustrating differences in testing strategies and preferences for alternative versus traditional approaches.

**Figure 5:**
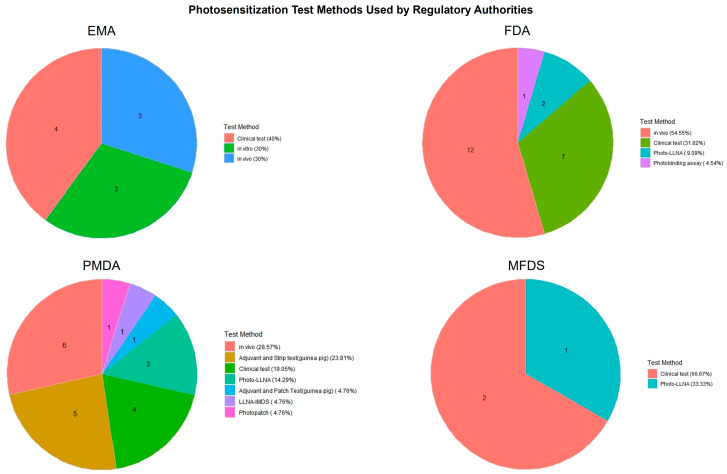
Distribution of photosensitization test methods used in pharmaceutical approvals by country. This figure highlights the diversity of test types adopted for photosensitization assessment across the EMA, FDA, PMDA, and MFDS, indicating variation in clinical, in vivo, and alternative testing practices.

Figure 4 and Figure 5 present a comparative overview of phototoxicity and photosensitization test methods employed by four major regulatory agencies: the EMA, FDA, PMDA, and MFDS. These pie charts illustrate how each country distributes its use of test strategies for photosafety assessments based on approved pharmaceutical dossiers.

In phototoxicity evaluations (Figure 4), the 3T3 NRU test method was the most frequently applied across all agencies, particularly by the PMDA (58.67%) and EMA (34.40%). The FDA showed more diversified usage, including the clinical test, UV spectrum analysis, and hemoglobin oxidation methods. The MFDS primarily relied on in vivo studies and the 3T3 NRU test, reflecting its recent emphasis on incorporating alternative methods.

In contrast, photosensitization testing (Figure 5) exhibited greater heterogeneity across agencies. The FDA and MFDS predominantly used in vivo tests and clinical tests, while the PMDA applied a wider range of methods, including in vivo tests, Photo-LLNA, and photopatch testing. The EMA utilized a more balanced mix of in vivo, in vitro, and clinical approaches. These results suggest differences in regulatory preferences and test accessibility, as well as varying degrees of reliance on traditional versus alternative methods.

**Figure 6:**
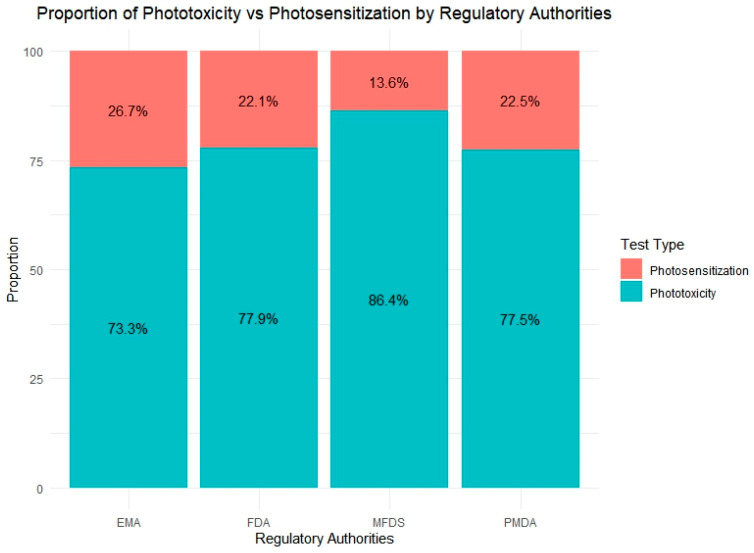
Distribution of photosafety test types submitted to four regulatory agencies (the EMA, FDA, PMDA, MFDS). Phototoxicity tests accounted for the majority of evaluations across all agencies, with the MFDS showing the highest proportion (86.4%). In contrast, the EMA reported the highest relative use of photosensitization tests (29%), indicating varying emphasis on test types among regulatory bodies.

Figure 6 provides a comparative overview of the relative proportion of phototoxicity and photosensitization tests conducted by four regulatory agencies: the EMA, FDA, PMDA, and MFDS. Across all countries, phototoxicity assessments represented the dominant form of photosafety testing, reflecting its broader regulatory adoption and the availability of validated test guidelines such as OECD TG 432.

Among the agencies, the MFDS reported the highest proportion of phototoxicity testing (86.4%), which may reflect its more recent enforcement of standardized safety requirements and a preference for phototoxicity evaluation during initial risk screening. In contrast, the EMA exhibited the greatest use of photosensitization tests (26.7%), indicating a relatively broader application or heightened regulatory interest in photoallergy assessments. The FDA and PMDA showed similar test ratios, with phototoxicity accounting for approximately 77% of their total photosafety submissions. These findings suggest that approaches to photosafety assessment vary among regulatory agencies.

## 4. Discussion

This study highlights the need for a harmonized and scientifically robust approach to photosafety evaluation by providing a comprehensive overview of current trends and challenges and by establishing a curated database of reference substances with a comparative analysis of validated in vitro methods against in vivo outcomes. Through the development of the PhotoChem Reference Chemical Database, we compiled detailed information on 251 substances, including phototoxic, non-phototoxic, and photosensitization classifications. Our database includes cosmetics [159], pharmaceuticals [48], and industrial and agricultural chemicals [54] for which photosafety assessment is required. It also integrates CAS numbers, physicochemical data, UV absorbance characteristics, and test results from the literature and drug approval dossiers across four major regulatory regions: the United States, Europe, Japan, and South Korea.

An evaluation of three key OECD in vitro test methods, namely TG 432, TG 495, and TG 498, revealed that all three in vitro test methods demonstrated high sensitivity and accuracy when compared to human and animal classifications. These results suggest strong potential for these methods to serve as alternatives to traditional animal-based testing. However, the notably lower specificity observed in TG 432 and TG 495 (54.3% and 63.6%, respectively) compared to TG 498 highlights an important limitation of current cell-based models. These methods are designed to maximize sensitivity to minimize false negatives, but this often leads to an increased incidence of false positives, reducing overall specificity. In contrast, TG 498, which employs reconstructed human epidermis models, demonstrated superior performance in both sensitivity and specificity despite having fewer data points. This suggests that 3D reconstructed human skin-based models may better mimic in vivo conditions, and their use in photosafety testing is likely to expand in future regulatory frameworks. The observed differences in specificity underscore the need for continued refinement and novel photosafety testing methods suited to particular regulatory contexts.

To further address these limitations and enhance the precision of in vitro models, quantitative structure–activity relationship (QSAR) models and artificial intelligence (AI)-based prediction systems may offer promising complementary approaches. QSAR models can prescreen compounds based on structural alerts and physicochemical properties, while AI models trained on multi source data including UV spectrum, historical assay results, and chemical fingerprints can provide integrated, quantitative predictions of phototoxic potential. Importantly, the PhotoChem DB developed in this study provides a robust foundation for building and validating such predictive models, suggesting that our study may contribute to the evolution of more accurate and human relevant non-animal assessment strategies.

An analysis of 106 pharmaceutical approvals from the FDA, PMDA, EMA, and MFDS revealed an increasing emphasis on phototoxicity evaluation across jurisdictions. A notable increase in test submissions to the MFDS was observed following the 2021 revision of its regulatory guidelines. Moreover, the distribution of test method preferences varied by country, with the 3T3 NRU and ROS assays commonly used for phototoxicity evaluation, while photo-LLNA and guinea pig assays were frequently applied for photosensitization.

Together, the curated database and comparative method analysis reinforce the importance of developing scientifically validated and internationally aligned nonanimal testing strategies. These findings also demonstrate the value of integrating physicochemical and UV-reactive properties to enhance predictive toxicology and support regulatory decision making.

## 5. Conclusions

In this study, we assessed the phototoxic potential of a range of chemical compounds using the 3T3 Neutral Red Uptake Phototoxicity Test (3T3 NRU PT), along with other validated in vitro methods. Several test substances exhibited significant responses upon UVA exposure, underscoring the importance of early stage photosafety screening, particularly for substances likely to be simultaneously exposed to skin and light. The 3T3 NRU PT method proved to be a reliable and reproducible tool for identifying phototoxicity, supporting its continued use in safety assessments. When benchmarked against human and animal data, TG 495 and TG 498 also showed excellent predictive performance, further validating the role of in vitro alternatives in regulatory applications. Furthermore, the PhotoChem Reference Chemical Database developed in this study serves as a valuable platform for advancing research, regulatory alignment, and method innovation. By combining chemical characteristics, UV absorption profiles, and cross-platform results, the database enables an integrated and data-driven analysis of photoreactivity risk.

Future research should aim to improve the precision of in vitro models, deepen mechanistic understanding, and adopt computational tools such as quantitative structure activity relationship (QSAR) models and artificial intelligence-based prediction systems. These approaches will facilitate scientifically rigorous and ethically responsible photosafety evaluation while reducing reliance on animal testing. In addition, the PhotoChem DB is expected to evolve as a dynamic, expandable resource by incorporating newly identified reference compounds, mechanistic data, and test results. Its continued development will also support the creation and validation of QSAR and AI-driven models, contributing to more precise, efficient, and human relevant safety assessments.

## Figures and Tables

**Table 1:** Phototoxicity and Photoallergy test methods.

Test Category		Test Strategy	Method
Phototoxicity	In vitro	In vitro 3T3 Neutral Red Uptake Phototoxicity Test (OECD TG 432)	Cell viability before and after UV exposure
		The Reactive Oxygen Species (ROS) Assay (OECD TG 495)	Measurement of ROS generation after light exposure, potentially causing cellular damage
		The Reconstructed Human Epidermis Phototoxicity Test (OECD TG 498)	Tissue viability test on reconstructed human epidermis after chemical exposure with or without UV exposure
	In vivo	Pigmented rats	Assessment of photoirritation responses in the skin and eyes after systemic administration of chemicals with or without UV exposure
		The Guinea Pig phototoxicity Test	Assessment of photoirritation responses in the shaved skin of guinea pigs after topical administration of chemicals with or without UV exposure
Photoallergy	In vivo	The Photo-LLNA (Local Lymph Node Assay)	A modified LLNA protocol measuring increased lymph node cell proliferation after UV exposure
		The Guinea Pig Photoallergy Test	A modified version of the Guinea Pig Maximization Test (GPMT) designed to evaluate allergic responses following UV sensitization

Abbreviations: TG = Test Guideline; ROS = Reactive Oxygen Species; LLNA = Local Lymph Node Assay; GPMT = Guinea Pig Maximization Test.

**Table 2:** Criteria for phototoxicity prediction according to OECD Test Guidelines.

Test Method	Prediction of Phototoxicity
OECD TG 432 [4] 3T3 Neutral Red Uptake Phototoxicity Test	Classification as phototoxic was assigned when the Photoirritation Factor (PIF) was ≥5 or the Mean Photo Effect (MPE) was ≥0.15.
OECD TG 495 [5] Reactive Oxygen Species Assay	Positive results were determined by the significant generation of reactive oxygen species (ROS) following UV exposure compared to non-irradiated controls.
OECD TG 498 [6] Reconstructed Human Epidermis Phototoxicity Test	Phototoxic if the UVA-exposed group(s) exhibit a 30% decrease in viability relative to the corresponding dark-exposed group.

**Table 3:** List of reference chemicals in the PhotoChem DB.

No.	Chemical	CAS No.	Physical State	Molar Extinction Coefficient (M^1^ cm^−1^)	UV λ_max_	Human	Animal	TG432 (3T3)	TG495 (ROS)	TG498 (RhE Model)	Others	References
1	Chlorpromazine hydrochloride	69-09-0	Solid	30,000–32,000	254	PT	PT	PT	PT	PT		[7,8,9,10,11,12,13,14]
2	Promethazine	60-87-7	Solid	21,000	250	PT	PT	PT		PT		[10,13,15,16,17,18,19,20]
3	5-Methoxypsoralen (5-MOP or Bergapten)	484-20-8	Solid	15,000	305	PT	PT	PT		PT		[8,13,15,16,19,20,21,22,23]
4	8-Methoxypsoralen (8-MOP or Methoxsalen)	298-81-7	Solid	18,400	300	PT	PT	PT	PT	PT		[8,9,11,13,14,15,16,18,19,22,23,24,25]
5	Para-aminobenzoic acid (PABA)	150-13-0	Solid	14,000	268	PS (photopatch)		NPT	NPT	NPT		[11,13,14,15,16,20,21,26,27]
6	Benzophenone-3	131-57-7	Solid	1150	288	PT	NPT	PT		NPT		[13,15,16,19,20,21,26]
7	Octyl methoxycinnamate	5466-77-3	Liquid	22,500	310	PT/NPT	NPT	NPT	NPT	NPT		[12,13,14,15,19,26]
8	Ecamsule (Mexoryl SX)	92761-26-7	Solid	32,000	344	NPT		NPT		NPT		[12,13,15,16]
9	6-Methylcoumarin	92-48-8	Solid	9500	326	PT	PT	PT	PT	NPT		[11,13,14,15,19,20,21,23,28,29]
10	4-tert-Butyl-3-methoxy-2,6-dinitrotoluene(Musk ambrette)	83-66-9	Liquid	NA	NA	PT	NPT	PT		NPT		[12,13,15,16,19,20,30]
11	Demeclocycline hydrochloride	64-73-3	Solid	13,400	270	PT	PT	PT		NPT		[8,12,13]
12	Bithionol	97-18-7	Solid	20,000	258	PT	PT	PT	PT	PT		[11,12,13,14,15,19,20,21,22,31]
13	Bergamot oil	8007-75-8	Liquid	NA	NA	PT	PT	PT		PT		[8,13,15,16,19,20,23,30,31,32]
14	Acridine hydrochloride	17784-47-3	Solid	11,800	355	PT	PT	PT	PT	PT		[12,13,14,20]
15	Anthracene	120-12-7	Solid	19,300	251	PT	PT	PT	PT	PT		[12,13,14,15,19,20,23]
16	Neutral red	553-24-2	Solid	8000	530	PT	PT	PT	PT	PT		[11,13,15,19,20,21,30]
17	Tetracyline	60-54-8	Solid	27,000	276	-	PT	-	PT	PT		[13,14,21]
18	Penicillin G	61-33-6	Solid	1700	264	NPT	NPT	NPT		NPT		[10,13,15,19,30]
19	Sodium lauryl sulfate (SDS)	151-21-3	Solid	NA	NA	NPT	NPT	NPT	NPT	NPT		[8,10,11,12,13,14,15,18,19,20,21,23,29,30]
20	Octyl salicylate	118-60-5	Liquid	900–1500	307	NPT	NPT	NPT	NPT	NPT		[12,13,14,15,30,32]
21	Benzylidene Camphor Sulfonic Acid	56039-58-8	Solid	28,000	310	NPT	NPT	NPT				[12,13]
22	4-Methylbenzylidene camphor (3-(4-Methylbenzylidene)camphor)	36861-47-9	Solid	27,000	303	PT/NPT	NPT	NPT	NPT	NPT		[10,12,13,15,19,30]
23	Benzalkonium chloride	139-07-1	Liquid	NA	NA		NPT	NPT		NPT		[15,33]
24	Acridine	260-94-6	Solid	11,800	355	PT	PT	PT		PT		[14,15,19,20,23,30]
25	Amiodarone	1951-25-3	Solid	25,000	242	PT	PT	PT		PT		[11,12,15,19,20]
26	Chlorhexidine dihydrochloride	3697-42-5	Solid	10,000	255		NPT	NPT				[11,20]
27	Chlorpromazine	50-53-3	Solid	3500–4500	256	PT	PT	PT		PT		[15,16,17,19,20,29,30,34]
28	Demeclocycline	127-33-3	Solid	1200	350	PT	PT	PT				[19,20]
29	Fenofibrate	49562-28-9	Solid	9800	290–310	PT		PT	PT			[8,14,19,20]
30	Furosemide	54-31-9	Solid	2300	273	PT		PT/NPT	PT			[8,9,14,19,20,29,35]
31	Hexachlorophene	70-30-4	Solid	12,000–14,000	270–290	PT/NPT	NPT	NPT	PT			[14,15,17,19,20,31,36]
32	Ketoprofen	22071-15-4	Solid	2000–3000	260–280	PT	PT/NPT	PT	PT			[8,9,12,14,19,20]
33	Nalidixic acid sodium salt	3374-05-8	Solid	5000–7000	270–280	PT	PT	PT	PT			[14,20]
34	Nalidixic acid	389-08-2	Solid	7500	276	PT	PT	PT	PT			[14,19,20]
35	Norfloxacin	70458-96-7	Solid	8000–10,000	277–278	PT	PT	PT	PT			[9,14,15,19,20,35]
36	Ofloxacin	82419-36-1	Solid	8000–10,000	293	PT	PT	PT	PT			[8,14,19,20,29]
37	Penicillin G sodium salt	69-57-8	Solid	1000–2000	230–250	NPT	NPT	NPT	NPT			[8,11,20,21]
38	Protoporphyrin IX	553-12-8	Solid	80,000	400–430	PT	PT	PT		PT		[15,19,20]
39	Protoporphyrin IX disodium salt	50865-01-5	Solid	80,000–90,000	405–410	PT	PT	PT				[12,20]
40	Xantryl(Rose Bengal (sodium salt)(1:2))	632-69-9	Solid	70,000	550	PT	NPT	PT	PT	PT		[11,14,20,21,23]
41	Tiaprofenic acid	33005-95-7	Solid	4000–5000	270	PT	PT	PT				[8,19,20]
42	Benzylidene camphor	15087-24-8	Solid	22,000–24,000	300	NPT		NPT				[12]
43	2-(Methylthio)phenol(o-(Methylthio)-phenol)	1073-29-6	Liquid	NA	NA			PT				[32,37]
44	Polyacrylamidomethyl benzylidene camphor	113783-61-2	Solid			NPT		NPT				[12]
45	Sulisobenzone (Benzophenone-4)	4065-45-6	Solid	20,000–30,000	290–360	NPT	NPT	NPT	NPT	NPT		[9,11,12,14,15,19,29,35]
46	Methyl-2-[(3,5,5- trimethylhexylidene) amino]benzoate	67801-42-7	Solid	NA	NA	NPT		PT		NPT		[32,37]
47	Promethazine hydrochloride	58-33-3	Solid	21,000	257	PT		PT		PT		[8,11,12,14,21]
48	Lime oil	8008-26-2	Liquid		315	PT	PT					[19,31,38]
49	Lemon oil	8008-56-8	Liquid			PT	PT	PT/NPT		PT		[15,31,33]
50	Orange oil	8028-48-6	Liquid			PT	PT	PT/NPT		PT		[15,31,33]
51	Rue oil	8014-29-7	Liquid			PT	PT					[22,31]
52	Cumin oil	8014-13-9	Liquid			PT	PT					[22,31]
53	Angelica root oil	8015-64-3	Liquid			PT	PT					[22,31]
54	Methylene blue	61-73-4	Liquid	74,000	665	PT						[31]
55	Eosin Y Disodium	17372-87-1	Solid	44,000	515	PT						[31]
56	Disperse blue 35	12222-75-2	Solid	NA	450–600	PT						[31]
57	Bengal rose (Rose bengale)	11121-48-5	Solid	>5000	500–600	PT	PT	PT		PT		[10,13,15,19,31]
58	3,3′,4′,5-Tetrachlorosalicylanilide (TCSA)	1154-59-2	Solid	NA	NA	PT	PT	PT				[11,19,31]
59	Tribromsalan(3,5,4′-Tribromosalicylanilide,TBS)	32055	Solid	NA	NA	NPT						[31]
60	Buclosamide(n-Butyl-4-chloro-2-hydroxybenzamide)	575-74-6	Solid			PT						[19,31]
61	Doxycycline hydrochloride	10592-13-9	Solid	20,000	270–280	PT	PT	PT	PT	PT		[9,11,14,19]
62	Tetracycline hydrochloride	64-75-5	Solid	10,000	270–280	PT	PT/NPT	PT		PT/NPT		[11,13,15,19,23]
63	Piroxicam	36322-90-4	Solid	4000	350	PT	PT	NPT	PT			[11,14]
64	Cinnamaldehyde	104-55-2	Liquid	6000	280	PS	NPT	NPT		NPT		[11,15,25,39]
65	L-Histidine	71-00-1	Solid	13,000	210	NPT	NPT	NPT	weak PR	NPT		[8,11,12,14,15,19,21]
66	Thiocarbamide(Thiourea)	62-56-6	Solid	7000	210	PS		NPT				[11,19]
67	Carprofen	53716-49-7	Solid	2000	254	PT	PT					[17,19]
68	Fenticlor	97-24-5	Solid	1000	250		PT					[17,19]
69	7-Acetyl-1,1,3,4,4,6- hexamethyltetra Hydronaphthalene	21145-77-7	Solid				PT					[22]
70	Galaxolide(4,6,6,7,8,8-Hexamethyl-1,3,4,6,7,8-hexahydrocyclopenta[g]isochromene)	1222-05-5	Liquid	NA	NA		PT					[22]
71	Celestolide(4-Acetyl-6-tert-butyl-1,1-dimethylindane)	13171-00-1	Solid	NA	NA		NPT					[22]
72	Phantolide	15323-35-0	Solid	NA	NA		NPT					[22]
73	Versalide	88-29-9	Solid	NA	NA		NPT					[22]
74	Ichthammol	8029-68-3	Liquid				PT	PT		PT		[15,40]
75	5-Aminolevulinic acid	106-60-5	Solid	28,000	400		PT	PT		PT		[15]
76	7-Methylcoumarin	2445-83-2	Solid	NA	NA		PT	PT		PT		[15]
77	Tetrachlorosalicylanilide	2018517	Solid	NA	NA		PT	PT		PT		[15]
78	Deterpenated lemon (-)			NA	NA		PT/NPT	PT/NPT		PT		[15,41]
79	Litsea cubeba oil	68855-99-2	Liquid	NA	NA		NPT	PT		NPT		[15,41]
80	ichthyolic acid, sodium salt	1340-06-3	Solid	NA	NA		NPT	PT		NPT		[15]
81	Avobenzone	70356-09-1	Solid	30,000–40,000	320–400	PT/NPT	NPT	PT	PT	NPT		[10,14,15,23,28,37,39,41,42,43,44]
82	Dimethyl sulfoxide	67-68-5	Liquid	NA	NA		NPT	NPT		NPT		[15]
83	Ethanol	64-17-5	Liquid	NA	NA		NPT	NPT		NPT		[15,18]
84	Eucalyptus oil	8000-48-4	Liquid	NA	NA		NPT	NPT		NPT		[15]
85	Coumarin	91-64-5	Solid	16,000–18,000	275	NPT	NPT	NPT				[8,15]
86	Titanium (IV) oxide	13463-67-7	Solid	~100,000	320–350					NPT		[15,39,43]
87	Cadmium sulfide	1306-23-6	Solid	58,000	450			PT		NPT		[7]
88	Cadmium selenide	1306-24-7	Solid	~100,000	560			NPT		NPT		[7]
89	Mercury(II) sulfide	1344-48-5	Solid					NPT		NPT		[7]
90	Chromium oxide	11118-57-3	Solid	10,000–40,000	visible region			NPT		NPT		[7]
91	Carbazole	86-74-8	Solid	6000–7000	290			PT		PT		[7]
92	Cobalt aluminum oxide	13820-62-7	Solid		500–700			NPT		NPT		[7]
93	Benoxaprofen	51234-28-7	Solid		320	PT	PT					[19]
94	Naproxen	22204-53-1	Solid	19,300	330	PT	PT					[19]
95	Suprofen	40828-46-4	Solid		244	PT						[19]
96	Triclosan	3380-34-5	Solid	4200	280	PT						[19]
97	Ciprofloxacin	85721-33-1	Solid	33,000	278	PT	PT					[19,25]
98	Fleroxacin	79660-72-3	Solid	25,000–35,000	278							[19]
99	lomefloxacin	98079-51-7	Solid	27,000–30,000	287	PT	PT					[8,19]
100	Bergaptol	486-60-2	Solid		290–320						NPT (V79 micronucleus assay)	[45]
101	Isopsoralen	523-50-2	Solid	15,000–20,000	300–320	PT						[8,19]
102	4,5′,8-Trimethylpsoralen (trioxsalen)	3902-71-4	Solid	10,000–20,000	300–320	PT						[19]
103	5-Fluorouracil	51-21-8	Solid	8200	266			NPT	NPT			[9,14,24,35,46,47]
104	Amiodarone hydrochloride	19774-82-4	Solid		290–350	PT	PT	PT	PT			[9,14,19,35]
105	Diclofenac sodium	15307-79-6	Solid	11,000–13,000	276	PT	PT	PT	PT			[9,14,19,35,48]
106	Levofloxacin	100986-85-4	Solid	16,000	292	PT	PT	PT	PT			[14,25,49,50,51]
107	Omeprazole	73590-58-6	Solid	18,000–22,000	302	PT		PT/NPT	PT			[9,14,35,49,52]
108	Quinine hydrochloride	130-89-2	Solid	5460	350	PT	NPT	PT	PT			[9,14,19,35,53]
109	Rosiglitazone	122320-73-4	Solid					PT				[14]
110	Bumetrizole	729335	Solid		300–400			NPT	NPT			[14]
111	Camphor sulfonic acid	1450959	Solid					NPT	NPT			[14]
112	Cinnamic acid	140-10-3	Solid	8000–10,000	260			NPT	Weak PT			[14]
113	Drometrizole	2440-22-4	Solid		290–320			NPT	NPT			[14]
114	Octrizole	3147-75-9	Solid		290–320			NPT	NPT			[14]
115	2-(2H-Benzotriazol-2-yl)-6-dodecyl-4-methylphenol	125304-04-3	Liquid		290–400			NPT				[14]
116	Aspirin	50-78-2	Solid	15,000–20,000	265			NPT	NPT			[14,35]
117	Benzocaine	34584	Solid	9000–11,000	260			NPT	NPT			[35]
118	Erythromycin	114-07-8	Solid	8000–10,000	230	NPT	NPT	NPT	NPT			[35,54,55]
119	Penicillin G potassium	113-98-4	Solid		203			NPT	NPT			[14,35]
120	Phenytoin	57-41-0	Solid	4500–6500	254			NPT	Weak PT			[14,35]
121	Chlorhexidine	55-56-1	Solid	1200–1400	260	NPT			Weak PT/NPT			[8,14,19]
122	Octyl methacrylate	93878	Liquid		210–220			NPT	NPT			[14]
123	Ethyl vanillin	121-32-4	Solid	11,000–13,000	270	(inconclusive)	NPT	PT		NPT		[32,37]
124	Vanillin isobutyrate	20665-85-4	Liquid		270–280	(inconclusive)	NPT	PT		NPT		[32,37]
125	Methyl 2,4-dihydroxy-3-methylbenzoate	33662-58-7	Solid		270–300	(inconclusive)	NPT	PT		PT		[32,37]
126	10H-Phenothiazine	92-84-2	Solid		255–265		PT	PT		PT		[32,56]
127	4-Acetoxy-3-ethoxybenzaldehyde	72207-94-4	Solid		250–300	(inconclusive)		PT		NPT		[32,37]
128	1-phenyl-3-(4-propan-2-ylphenyl)propane-1,3-dione	63250-25-9	-		250–300	PT						[19,26]
129	Minocycline	10118-90-8	Solid	7700	350	NPT						[19]
130	Chlordiazepoxide	58-25-3	Solid		250–260		PT					[19]
131	Diflunisal	22494-42-4	Solid		252		PT					[19]
132	Griseofulvin	126-07-8	Solid		290		PT					[19]
133	Chloroquine	19851	Solid	7000–12,000	260		NPT					[19]
134	Chlortetracycline	57-62-5	Solid	8500–12,000	270		NPT					[19]
135	chlorothiazide	58-94-6	Solid		260		NPT					[19]
136	Clomocycline	1181-54-0	-		270–280		NPT					[19]
137	Cyclamic acid	100-88-9	Solid		250–260		NPT					[19]
138	Fenoprofen	29679-58-1	Solid		270–280		NPT					[19]
139	Flurbiprofen	5104-49-4	Solid		247–260		NPT					[19]
140	Ibuprofen	15687-27-1	Solid		264		NPT					[19]
141	Indoprofen	31842-01-0	Solid		250–280		NPT					[19]
142	Methacycline	914-00-1	Solid		270–280		NPT					[19]
143	Oxytetracycline	79-57-2	Solid		270		NPT					[19]
144	Tolbutamide	64-77-7	Solid		230–240		NPT					[19]
145	Vanillin propylene glycol acetal	68527-74-2	Liquid		270–280	(inconclusive)		PT		NPT		[19,37]
146	4′-Hydroxy-3′-methoxyacetophenone(acetovanillone)	498-02-2	Solid		280–300	(inconclusive)		PT		NPT		[19,37]
147	Vanillin	121-33-5	Solid	15,000–18,000	270			PT		NPT		[37]
148	2-Methoxycinnamaldehyde	1504-74-1	Solid		270–290	(inconclusive)		PT		NPT		[37]
149	5-Methylquinoxaline	13708-12-8	Liquid		260–300	(inconclusive)		PT		PT		[37]
150	Capmatinib	1029712-80-8	Solid				PS	PT				[57]
151	Fosdenopterin hydrobromide	2301083-34-9	Solid				PT	PT				[58]
152	Berdazimer sodium	1846565-00-1	gel								NPT(UV spectrum)	[59]
153	Tretinoin	302-79-4	Solid							NPT		[60]
154	Sabizabulin	1332881-26-1	Solid					PT				[61]
155	Rezafungin acetate	1631754-41-0	Solid				PT	PT				[62]
156	Viloxazine hydrochloride	35604-67-2	Solid	56.62	290						NPT(UV spectrum)	[63]
157	ESTRADIOL	50-28-2	Solid				NPT					[64]
158	Oxybutynin	5633-20-5	Solid				NPT					[65]
159	Darolutamide	1297538-32-9	Solid	23,100–22,500	290–320			NPT			NPT(UV spectrum)	[66,67]
160	Berotralstat Hydrochloride	1809010-52-3	Solid					NPT				[68]
161	Esflurbiprofen	51543-39-6	Solid					NPT				[69]
162	Fosravuconazole L-lysine ethanolate	914361-45-8	Solid					NPT				[70]
163	Quizartinib	950769-58-1	Solid					NPT				[71]
164	Entrectinib	1108743-60-7	Solid				PT	PT				[72]
165	Filgotinib Maleate	1802998-75-9	Solid				NPT	PT				[73]
166	Riociguat	625115-55-1	Solid		290–720		NPT	PT/NPT				[74]
167	Dabrafenib Mesylate	1195768-06-9	Solid					PT				[75]
168	Encorafenib	1269440-17-6	Solid					PT				[76]
169	Trametinib Dimethyl Sulfoxide	1187431-43-1	Solid	>1000	290–700		PT	PT				[77,78]
170	Suvorexant	1030377-33-3	Solid				NPT					[79]
171	Molnupiravir	2349386-89-4	Solid	>1000	290–700				NPT		NPT(UV spectrum)	[79]
172	Gefapixant	1015787-98-0	Solid		290–350			NPT			NPT(UV spectrum	[80]
173	Bictegravir sodium	1807988-02-8	Solid				NPT	PT				[81]
174	Lenacapavir sodium	-	Solid					NPT				[82]
175	Doravirine	1338225-97-0	Solid				NPT	NPT				[83]
176	Eltrombopag olamine	496775-62-3	Solid			NPT	NPT	PT				[84]
177	Bisoprolol	66722-44-9	Solid				PT/PS					[85]
178	Binimetinib	606143-89-9	Solid				PT	PT				[86]
179	Ibandronate Sodium	138844-81-2	Solid				NPS	PT				[87]
180	Ledipasvir	1256388-51-8	Solid				NPT					[88]
	Sofosbuvir	1190307-88-0	Solid									
181	Dolutegravir sodium	1051375-19-9	Solid			NPT						[89]
182	Sofpironium Bromide	1628106-94-4	Solid	>1000							NPT(UV spectrum)	[90]
183	Isavuconazonium sulfate	946075-13-4	Solid					NPT				[91]
184	Ribavirin	36791-04-5	Solid				NPT	PT				[92]
185	Fosnetupitant Chloride Hydrochloride	1643757-72-5	Solid					PT				[93]
186	Risdiplam	1825352-65-5	Solid					NPT				[94]
187	Opicapone	923287-50-7	Solid				NPS	NPT				[95]
188	Difamilast	937782-05-3	Solid				NPT/NPS	NPT				[96]
189	Sirolimus((-)-Rapamycin)	53123-88-9	Solid				NPS	NPT				[97]
190	Momelotinib	1056634-68-4	Solid					NPT				[98]
191	Siponimod	1230487-00-9	Solid	3309	260			NPT				[99]
192	Asciminib	1492952-76-7	Solid				PT/PS	PT				[100,101]
193	Artemether	71963-77-4	Solid				PT					[102]
	Lumefantrine	82186-77-4	Solid									
194	Cabotegravir	1051375-10-0	Solid	2670–20,800	257						NPT(UV spectrum)	[103]
195	Baricitinib	1187594-09-7	Solid	>1000	290–329			NPT			PT(UV spectrum)	[104]
196	Anamorelin	249921-19-5	Solid	4958	291						NPT(UV spectrum)	[105]
197	Delgocitinib	1263774-59-9	Solid				NPT/NPS	NPT				[106]
198	Bedaquiline fumarate	845533-86-0	Solid				NPT/NPS	PT				[107]
199	Nintedanib	656247-17-5	Solid					PT				[108]
200	Pazopanib	444731-52-6	Solid					NPT				[109]
201	Roxadustat	808118-40-3	Solid					NPT				[110]
202	Selexipag	475086-01-2	Solid			mild PT		PT				[111]
203	Fulvestrant	129453-61-8	Solid					NPT				[112]
204	Degarelix acetate	934016-19-0	Solid					NPT				[113]
205	Remimazolam besilate	1001415-66-2	Solid					NPT				[114]
206	Selumetinib	606143-52-6	Solid	11,786	290			NPT				[115]
207	Pimitespib	1260533-36-5	Solid					NPT				[116]
208	Tirabrutinib hydrochloride	1439901-97-9	Solid					NPT				[117]
209	Vemurafenib	918504-65-1	Solid		240–450		NPT	PT				[118,119]
210	Avibactam Sodium	1192491-61-4	Solid					NPT				[120]
	Ceftazidime pentahydrate	78439-06-2	Solid									
211	Dasatinib	302962-49-8	Solid				NPT	PT				[121]
212	Diclofenac Etalhyaluronate Sodium	1398396-25-2	viscous					NPT				[122]
213	Aripiprazole	129722-12-9	Solid					NPT				[123]
214	Terbinafine hydrochloride	78628-80-5	Solid			NPT	NPT/NPS					[124]
215	Methyl Salicylate	119-36-8	liquid				NPT/NPS					[125]
216	Ivermectin	70288-86-7	Solid		<290						UV spectrum	[126]
217	Docosanol	661-19-8	solid				NPT/NPS					[127]
218	Butenafine hydrochloride	101827-46-7	solid				NPT/NPS					[128]
219	Diclofenac sodium	15307-79-6	solid				NPT/NPS					[129]
220	Avobenzone	70356-09-1	Solid				NPT/NPS					[130]
	Ecamsule	92761-26-7	Solid	32,000	344							
	Octocrylene	6197-30-4	liquid									
221	Trifarotene	895542-09-3	solid				NPT					[131]
222	Adapalene	106685-40-9	solid							NPT		[132]
223	TIRBANIBUlin 1%	897016-82-9	solid				NPT/NPS					[133]
224	Tazarotene	118292-40-3	solid				NPT/NPS					[134]
225	Tapinarof	79338-84-4	solid				NPT					[135]
226	SULFACETAMIDE SODIUM	127-56-0	solid			NPT						[136]
227	SULCONAZOLE NITRATE	61318-91-0	solid			NPT						[137]
228	Spinosyn A	131929-60-7	solid				NPT					[137]
	Spinosyn D	131929-63-0	solid									
229	Sofpironium bromide	1628106-94-4	solid/gel								NPT(UV spectrum)	[90]
230	Ruxolitinib	941678-49-5	oil			NPT	NPT					[138]
231	Roflumilast	162401-32-3	solid			NPT		NPT				[139]
232	Pimecrolimus	137071-32-0	solid			NPT	PT					[140]
233	Penciclovir	39809-25-1	solid			NPT	NPT				NPT(UV spectrum	[141]
234	Ozenoxacin	245765-41-7	solid			NPT	NPT					[142]
235	Oxymetazoline hydrochloride	151615	solid				NPT					[143]
236	Alitretinoin	1241893	solid							PT	PT(hemoglobin assay, histidine assay)	[144]
237	Aminolevulinic acid hydrochloride	1297222	solid			PS	PT					[145]
238	Benzoyl peroxide	94-36-0	solid							NPT	PT(hemoglobin assay, histidine assay)	[146]
239	Birch triterpenes	botanical drug	gel			NPS	NPS	NPT				[147]
240	Brimonidine tartrate	70359-46-5	solid				NPT					[148]
241	Bexarotene	153559-49-0	solid							NPT	PT(hemoglobin assay, histidine assay)	[146]
242	Capsaicin	404-86-4	solid				NPT					[149]
243	Ciclopirox	29342-05-0	solid				NPT					[150]
244	Clascoterone	19608-29-8	solid								NPT(UV spectrum)	[151]
245	Dapsone	80-08-0	solid				NPT					[152]
246	Econazole nitrate	24169-02-6	solid				NPT					[153]
247	Efinaconazole	164650-44-6	solid				NPT					[154]
248	Fluorouracil	51-21-8	solid		265–266		NPT					[155]
249	Glycopyrronium tosylate	1883451-12-4	solid								NPT(UV spectrum)	[156]
250	Ivermectin	70288-86-7	solid				NPT					[157]
251	Luliconazole	187164-19-8	solid				NPT/NPS					[158]

Abbreviations: PT = Phototoxic; NPT = Non-Phototoxic; PS = Photosensitization. Of the 251 reference chemicals listed above, the majority were solids (86.9%), followed by liquids (10.4%). Gels, viscous substances, and unknowns made up a minor proportion of the database.

**Table 4:** Predictive performance of in vivo phototoxicity tests compared with human patch tests.

		Animal	
		PT	NPT
**Human**	PT	40	4
	NPT	1	13
	Total (n)		58
	Sensitivity		90.9%
	Specificity		92.9%
	Accuracy		91.4%

Abbreviations: PT = Phototoxic; NPT = Non-Phototoxic; n = number of substances.

**Table 5:** Predictive performance of in vitro phototoxicity tests compared with human test results.

	TG432 (3T3 NRU)		TG495 (ROS Assay)		TG498 (Epiderm Model)	
	PT	NPT	PT	NPT	PT	NPT
**Human vs. in vitro phototoxic test results**						
PT	36	1	22		19	4
NPT	2	13		5		7
Total (n)		52		27		30
Sensitivity		97.3%		100%		82.6%
Specificity		86.7%		100%		100%
Accuracy		94.2%		100%		86.7%
**Animal vs. in vitro phototoxic test results**						
PT	38	1	17		23	2
NPT	16	19	4	7	2	19
Total (n)		74		28		46
Sensitivity		97.4%		100%		92.0%
Specificity		54.3%		63.6%		90.5%
Accuracy		77.0%		85.7%		91.3%

Abbreviations: PT = Phototoxic; NPT = Non-Phototoxic; TG = OECD Test Guideline.

## Data Availability

The data presented in this study are available from the corresponding author on request.

## Abbreviations

The following abbreviations are used in this manuscript:

OECD	The Organization for Economic Co-operation and Development
ICH	International Council for Harmonization of Technical Requirements for Pharmaceuticals for Human Use
FDA	U.S. Food and Drug Administration
PMDA	Japan Pharmaceuticals and Medical Devices Agency
EMA	European Medicines Agency
